# SSR and SRAP marker-based linkage map of *Vitis vinifera* L.

**DOI:** 10.1080/13102818.2014.907996

**Published:** 2014-07-14

**Authors:** Yinshan Guo, Hong Lin, Zhendong Liu, Yuhui Zhao, Xiuwu Guo, Kun Li

**Affiliations:** ^a^ Pomology Department, College of Horticulture, Shenyang Agricultural University,Shenyang, P.R. China

**Keywords:** *Vitis vinifera* L., genetic maps, SSR, SRAP

## Abstract

An F1 population was created by the cross ‘87-1’ × ‘9-22’. The female parent ‘87-1’ was an extremely early maturing cultivar with strong flavour. The male parent was an excellent breeding line producing large berries maturing late. The mapping population included 149 randomly chosen individuals. Molecular genetic map for each parent and the consensus map were constructed using simple sequence repeat and sequence-related amplified polymorphism markers by software JoinMap 3.0. The ‘87-1’ map covers a total length of 1272.9 cM distributed in 21 linkage groups and consists of 163 molecular markers with an average distance between adjacent markers of 8.9 cM. The ‘9-22’ map covers a total length of 1267.4 cM distributed in 20 linkage groups and consists of 158 molecular markers with an average distance between adjacent markers of 9.1 cM. The consensus map covers a total length of 1537.1 cM distributed in 21 linkage groups and one doublet and consists of 217 molecular markers with an average distance of 7.8 cM between adjacent markers. The length of the linkage groups is 69.8 cM on average. The map covers the 19 chromosomes of the *Vitis* genome and can lay a solid foundation for further studies such as quantative trait loci (QTL) mapping of correlated traits and marker-assisted selection.

## Introduction

Grapevine is an old and important economic crop, and is cultivated throughout the world. Up to 2011, the global harvested area of grapevine reached 7.0 million hectares, and the yield exceeded 69 million tons. As a major fruit crop, grape has been causing widespread concern in cultivar modification in many countries. However, grapevine has a series of characters including complicated genetic background, large plant body and long generation cycle, etc., which leads to the restriction of grape breeding. The construction of genetic linkage maps is a major part of genome research and is necessary for gene mapping, gene cloning and genome structure and function research. The construction of high-density genetic map assists the genetic analysis of important agronomic traits at the molecular level, and as a matter of course, could lay the foundation for marker-assisted selection.

With the development of molecular marker technology and the application of ‘Double Pseudo-Test Cross Theory’ in pomology genetic research, genetic-map construction studies have been experiencing great progress. Since the first *Vitis* genetic map was released,[1] many *Vitis* maps, including maps of *V. vinifera*,[2–4] *V. rupestris*,[5] *V. riparia* [6] and *V. champini* [7,8] have been constructed by grape researchers.

Previous maps were mainly based on dominant markers, such as random amplified polymorphic DNA (RAPD) and amplified fragment length polymorphism (AFLP).[1,9] The co-dominant simple sequence repeat (SSR) marker for *Vitis* was successfully developed in 1993,[10] and its conservatism and generality was then verified in 2000.[11] Subsequently, a lot of maps based on SSR markers have been successfully constructed.[3–8,12–17] SSR markers became one of the main molecular markers of *Vitis* genetic maps. What is more, a number of *Vitis* genetic maps were based on SSR marker together with other markers, such as Sequence Characterized Amplified Regions (SCAR),[18–20] expressed sequence tag-simple sequence repeat (EST-SSR) [21,22] and single nucleotide polymorphism (SNP).[16,22–25] And the combination of different markers enhanced the density and genome coverage of the maps.

The sequence-related amplified polymorphism (SRAP) marker is a type of dominant marker, which targets the coding sequences in the genome, especially the open reading frames (ORFs), so as to increase the relationship between amplification results and phenotype.[26] And SRAP has already been used in genetic diversity analysis,[27–29] genetic-map construction [8,30–32] and QTL detection.[33,34] A *V. amurensis* map based on SSR marker along with SRAP marker was constructed by Liu et al., [8] who found that SRAP markers could well fill the gaps between SSR markers and could also increase the genome coverage of the map. Here, we use both SSR and SRAP markers to construct a *V. vinifera* genetic linkage map, aiming to provide support for QTL mapping and marker-assisted selection in grape.

## Materials and methods

### Plant material

F1 population was obtained from the intraspecific cross ‘87-1’ × ‘9-22’. The female parent ‘87-1’, which is peculiar to China, is an extremely early maturing cultivar with strong flavour. Cultivar ‘9-22’ is a complex hybrid containing the genetic background of Italia, Rizamat and Moscato Blanco. The parent ‘9-22’ produces large berries with fragile pulp and high soluble solid content, but without any aroma. We randomly chose 149 individuals from the F1 population as the mapping population, along with the two parents, to perform molecular marker analysis and genetic-map construction. The experiment was carried out in the Molecular Biology Laboratory, Pomology, Shenyang Agricultural University. The mapping population and parents were all grown in the grape breeding nursery in Shenyang Agricultural University.

Young leaves were collected for DNA extraction, according to the modified cetyltrimethyl ammonium bromide (CTAB) method.[35]

### SSR markers and progeny genotyping

The primer pairs flanking microsatellite loci were from marker sets VMC (Vitis Microsatellite Consortium, managed through AGROGENE, Moissy Cramayel, France), VVS,[10] VVMD,[36,37] VrZAG,[38] VVI,[39] UDV [40] and Chr. [7] In total 247 SSRs were used for map construction.

Polymerase chain reactions (PCR) were done in 16 μL mixtures containing 10 ng of template DNA, 2.0 mmol/L MgCl_2_, 100 μmol/L dNTPs, 0.3 μmol/L of each primer, 1 × PCR buffer and 0.8 U of Taq polymerase. Optimal annealing temperatures were optimized for each primer pair, using gradient annealing temperatures (from 50 to 63 °C) programmed by a S1000TM Cycler, keeping all other conditions of the amplification protocol constant (4 min at 94 °C followed by 25 cycles of 1 min at 94 °C, 1 min at optimized annealing temperature, 1 min at 72 °C followed by a final step of 7 min at 72 °C). Amplification products were separated by 5% native polyacrylamide gel electrophoresis (PAGE) and visualized by silver staining.

### SRAP markers and progeny genotyping

SRAP primers were from Li and Quiros.[26] A small population consisting of six randomly chosen individuals was used for the selection of 144 primer pairs (Table 1). Finally, 30 primer pairs, which produced stable, clear and polymorphic bands, were selected for the amplification of the mapping population.

**Table 1.** Forward and reverse primer sequences used in SRAP analysis.

Forward primers	Reverse primers
me1 5′-TGAGTCCAAACCGGATA-3′	em1 5′-GACTGCGTACGAATTAAT-3′
me2 5′-TGAGTCCAAACCGGAGC-3′	em2 5′-GACTGCGTACGAATTTGC-3′
me3 5′-TGAGTCCAAACCGGAAT-3′	em3 5′-GACTGCGTACGAATTGAC-3′
me4 5′-TGAGTCCAAACCGGACC-3′	em4 5′-GACTGCGTACGAATTTGA-3′
me5 5′-TGAGTCCAAACCGGAAG-3′	em5 5′-GACTGCGTACGAATTAAC-3′
me6 5′-TGAGTCCAAACCGGTAG-3′	em6 5′-GACTGCGTACGAATTGCA-3′
me7 5′-TGAGTCCAAACCGGTTG-3′	em7 5′-GACTGCGTACGAATTATG-3′
me8 5′-TGAGTCCAAACCGGTGT-3′	em8 5′-GACTGCGTACGAATTAGC-3′
me9 5′-TGAGTCCAAACCGGTCA-3′	em9 5′-GACTGCGTACGAATTACG-3′
me10 5′-TGAGTCCAAACCGGAGG-3′	em10 5′-GACTGCGTACGAATTTAG-3′
me11 5′-TGAGTCCAAACCGGAGA-3′	em11 5′-GACTGCGTACGAATTTCG-3′
me12 5′-TGAGTCCAAACCGGAAA-3′	em12 5′-GACTGCGTACGAATTGTC-3′
me13 5′-TGAGTCCAAACCGGAAC-3′	em13 5′-GACTGCGTACGAATTGGT-3′
me14 5′-TGAGTCCAAACCGGACA-3′	em14 5′-GACTGCGTACGAATTCAG-3′
me15 5′-TGAGTCCAAACCGGACG-3′	em15 5′-GACTGCGTACGAATTCTG-3′
me16 5′-TGAGTCCAAACCGGACT-3′	em16 5′-GACTGCGTACGAATTCGG-3′
me17 5′-TGAGTCCAAACCGGCAT-3′	em17 5′-GACTGCGTACGAATTCCA-3′
me18 5′-TGAGTCCAAACCGGGAC-3′	em18 5′-GACTGCGTACGAATTGAT-3′
me19 5′-TGAGTCCAAACCGGGTA-3′	em19 5′-GACTGCGTACGAATTCAA-3′
me20 5′-TGAGTCCAAACCGGGGT-3′	em20 5′-GACTGCGTACGAATTCAT-3′
me21 5′-TGAGTCCAAACCGGCAG-3′	em21 5′-GACTGCGTACGAATTCTA-3′

The reaction system was 20 μL, including: 2.0 mmol/L MgCl_2_, 0.1 mmol/L dNTPs, 0.5 μmol/L primer, 10 ng of template DNA and 1.5 U of Taq DNA polymerase. The protocol for PCR amplification was: initial denaturation (5 min at 94 °C); denaturation (60 s at 94 °C) for five cycles; denaturation (60 s at 94 °C), annealing (60 s at 50 °C), extension (90 s at 72 °C), for 35 cycles; final extension (10 min at 72 °C). The amplification products were separated by electrophoresis in 7% polyacrylamide gels.

### Segregation analysis and map construction

For co-dominant loci, fully informative markers with four (ab × cd) or three segregating alleles (ef × eg), double heterozygous markers (hk × hk) and markers only segregating in the female parent (lm × ll) were used to construct the female map. Fully informative markers, double heterozygous markers and markers only segregating in the male parent (nn × np) were used to build the male map. For dominant loci, markers segregating in both parents (hk × hk) and markers only segregating in the female parent (lm × ll) were used to construct the female map; markers segregating in both parents (hk × hk) and markers only segregating in the male parent (nn × np) were used to construct the male map. All marker sets together yielded the integrated map. Parental maps were created with JoinMap 3.0.

LOD value was set from 3.0 to 5.0, and the maximum recombination value was 0.4. The recombination rate was converted into map distance (cM) by the Kosambi function.[41] Parental molecular genetic maps were drawn with the Mapchart 2.2 software.[42] The linkage groups were numbered according to IGGP (http://www.vitaceae.org) reference map.[12,19]

## Results and discussion

### Analysis of SSR and SRAP markers

Among the 247 SSR primer pairs, 75 produced low-quality bands or no bands, and then were discarded. For the other 172 primer pairs, 17 yielded full co-dominant information exhibiting four alleles (ab × cd), and 40 primer pairs segregated with three alleles (ef × eg). Four primer pairs produced bands segregating from both parents with the same size scored as double heterozygous markers following the pattern (hk × hk). Twenty-five markers only segregated in the female parent (lm × ll), and 27 markers only in the male parent (nn × np) (Table 2). The remaining 59 pairs showed a homozygous profile in both parents, resulting in a heterozygous rate of 65.6%. Thirty SRAP primer pairs produced a total of 139 polymorphic markers, among which 53 separated only in the female parent (maternal specific), 47 separated only in the male parent (paternal specific) and 39 separated in both parents (common markers, theoretically according to the separation of 3:1).

**Table 2.** Distribution of segregation types in the ‘87-1’ × ‘9-22’ population.

‘87-1’		‘9-22’		‘87-1’ and ‘9-22’				
1:1		1:1		1:1:1:1		1:1:1:1	1:2:1	Total
lm x ll (codominant)	lm x ll (dominant)	nn × np (dominant)	nn × np (codominant)	ab × cd	ef × eg	hk × hk (codominant)	hk × hk (dominant)	3:1
25	53	27	47	17	40	4	39	252

The X^2^ test showed that, for co-dominant markers, three paternal specific markers, two maternal specific markers and six common markers deviated from the Mendelian segregation ratio. The markers showing distorted segregation ratio were 9.7% of all co-dominant markers.

### Genetic mapping

To construct the maternal map, 178 molecular markers containing 86 co-dominant ones and 92 dominant ones were used. One hundred and sixty-three markers (83 co-dominant, 80 dominant) formed the map with a total length of 1272.9 cM (Table 3, Figure 1). These markers were distributed into 21 linkage groups. The mean distance between adjacent markers was 8.9 cM. The mean length of the linkage groups was 60.6 cM. The largest group was LG4 (133.1 cM), consisting of seven SSRs and five SRAPs. Compared with the international reference map [12,19] (the same below), our LG8 and LG13 were split into fractions because of weak linkage. The largest gap of 40.7 cM was found in LG17.

**Table 3.** Main characteristics of linkage groups in the integrated, the maternal ‘87-1’ and the paternal ‘9-22’ map.

	Consensus map			Map of female parent ‘87-1’			Map of male parent ‘9-22’		
LGs	Covered length (cM)	No. of markers	Gaps (>20 cM) number	Covered length (cM)	No. of markers	Gaps (>20 cM) number	Covered length (cM)	No. of markers	Gaps (>20 cM) number
1	51.7	8	0	46.3	3	1	51.7	7	0
2	19.2	3	0	19.2	3	0	12.4	2	
3	139.7	17	3	99.6	13	2	105.6	13	2
4	94.3	9	2	133.1	12	2	69.4	8	1
	63.4	5	1						
5	90.7	10	1	90.7	9	1	54.5	6	1
6	115	14	2	47.8	15	0	115	14	2
7	90.6	13	1	34.9	3	1	91.6	11	2
8	74.9	7	1	48.1	5	0	74.9	7	1
8 associated	16.2	4	0	12.8	3		15.6	3	0
9	137.9	17	2	95.6	10	2	153.6	11	4
10	62.4	15	1	94.5	11	1	62.8	13	1
11	9.7	2	0	9.7	2	0	–	–	–
12	79.2	16	0	97.1	10	1	84.7	13	0
13	40.7	5	1	28.7	2	1	40.7	5	1
13 associated	53.9	3	1	53.9	3	1	–	–	–
14	61.7	15	0	61.5	14	0	66	10	0
15	16.2	4	0	16.2	4	0	11	3	0
16	42	12	0	40.6	11	0	39.3	9	1
17	62.4	8	1	62.7	7	1	34.6	4	1
18	108	12	2	77.9	8	2	104.9	6	2
19	107.3	18	0	102	15	0	79.1	13	0
Total	1537.1	217	19	1272.9	163	16	1267.4	158	19
Average	69.8			60.6			63.3		

Figure 1. Linkage map of the integrated, the maternal ‘87-1’ and the paternal ‘9-22’ maps. The name of SRAP marker consists of three parts: ‘the combination of forward and reverse primers’ + ‘the origin of the amplified product’ + ‘the size of the product’. ‘F’, ‘M’ and ‘C’ denote that the product was from the female parent, the male parent or both parents, respectively, e.g. ‘m7e21F-121’ means a 121 bp product amplified in the female parent by primer combination me7em21.

**Figure:**
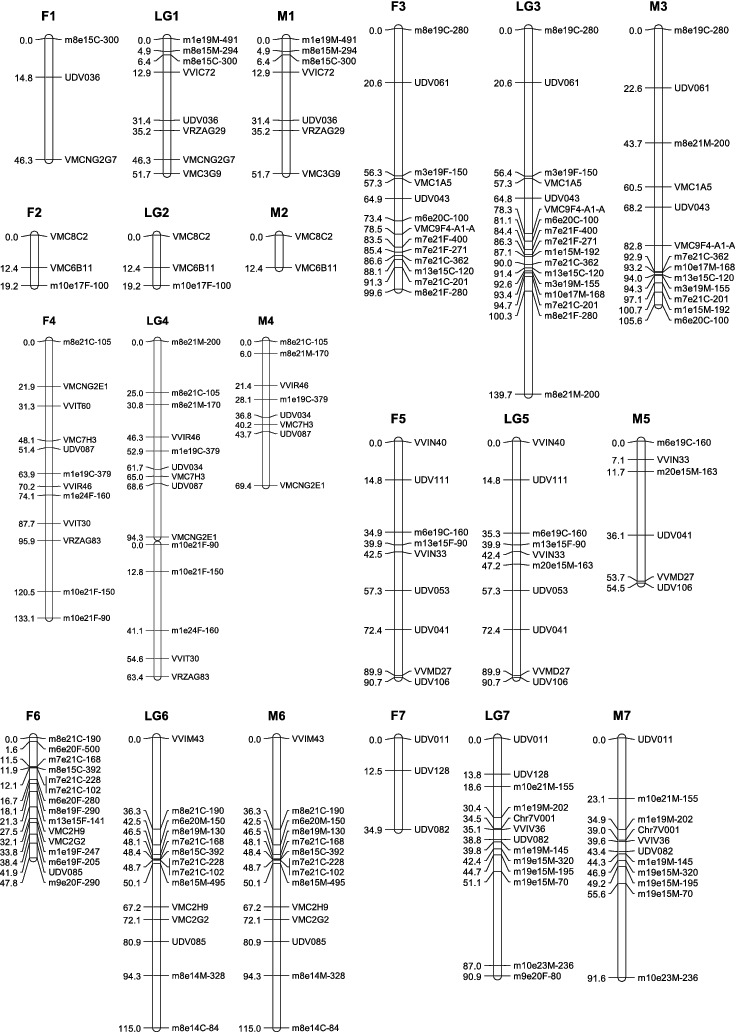

**Figure:**
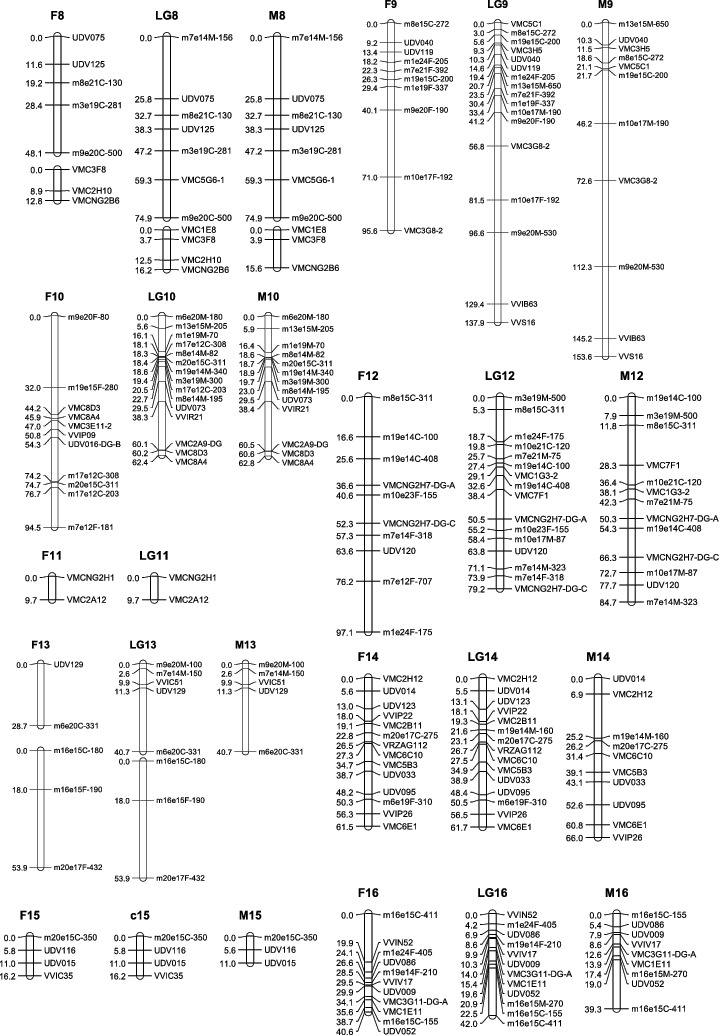

**Figure:**
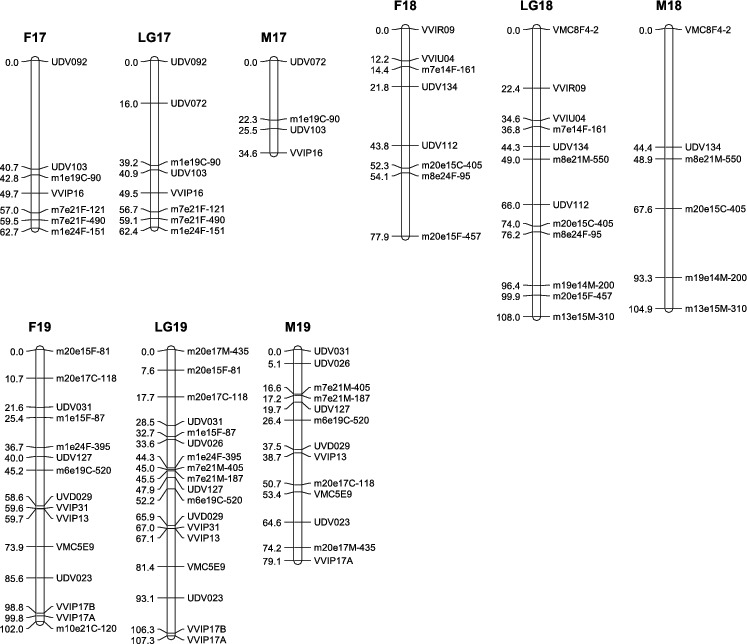


The paternal map was constructed based on 174 molecular markers containing 88 co-dominant ones and 86 dominant ones. One hundred and fifty-eight markers (76 co-dominant, 82 dominant) formed the map with a total length of 1267.4 cM (Table 3 and Figure 1). These markers allocated into 20 linkage groups. The mean distance between adjacent markers was 9.1 cM. The mean length of the linkage groups was 63.3 cM. The largest group was LG9 (153.6 cM), consisting of six SSRs and five SRAPs. Compared with the international reference map, our LG8 was split into fractions because of weak linkage. The largest gap of 44.4 cM was found in LG18. And LG11 has not been constructed.

All markers (252 markers) were gathered together for integrated map construction. Finally, 217 molecular markers formed 20 linkage groups and a doublet. The whole length of the map was 1537.1 cM, with an average distance between adjacent markers of 7.8 cM, and a mean linkage group length of 69.8 cM. The largest group was LG9 (153.6 cM), containing six SSRs and five SRAPs. The smallest group was LG11 (9.7 cM), containing only two markers. Compared with the reference map, LG4, LG8 and LG13 were all hereby split into two groups. There were two large gaps in LG3, which were between UDV016 and m3e19F-15 (35.8 cM), and between m8e21F-280 and m8e21M-200 (39.4 cM). Most of the co-dominant markers had the same colinearity with the two reference maps, except for the following differences, due to the anomalous map location of VMC2G2 and VMC2H9 in LG6, VMC3H5 and VMC5C1 in LG9 and VMC1E11 and VVIV17 in LG17. In addition, VVIB63 and VVS16 existing in LG15 of the reference maps, appeared in LG9 of our map.

### Final remarks

Genetic maps could help breeders to detect strong linkages and co-dominant inheritance, and could also affirm the number and position of genetic factors controlling quantitative characters.[43] Up to now, a number of *Vitis* genetic maps have been reported, referring to the cross within *V. vinifera*,[2,4,12,16] interspecific hybridization[6,19,13,24,44,45] and other subspecies.[5,7,8] What is more, several QTLs of important traits have been mapped.

In this study, the intraspecific population was created using ‘87-1’ and ‘9-22’ which were originated in China. The female parent ‘87-1’ is extremely early maturing and with strong flavour. The male parent ‘9-22’ is mid-late maturing and with large berry of no flavour. We constructed molecular genetic maps of high density. Although containing several gaps, the maps we constructed cover the 19 chromosomes of the *Vitis* genome and can be used for QTL mapping of correlated characters.

Marker distortion is a common phenomenon in the mapping process.[3,6,16] The discordance of parents, the chromosomal structure rearrangement, deletion, insertion and mutation could all lead to marker distortion in the next generation. In addition, other factors can also lead to marker distortion, such as a small mapping population as well as band missing as a result of PCR amplification or electrophoresis. The proportion of SSR markers with biased segregation observed in this study (9.7%) was lower than those reported by Grando et al. (22.4%) [3], Lowe and Walker (16%), [6] Troggio et al. (20.3%) [16] and Blasi et al. (11.3%), [7] but was almost the same with that reported by Doligez et al. (9.2%). [12]

In some genetic mapping studies, markers without serious distortion or markers which did not affect marker order along the linkage group were used for map construction.[2,17] Bradshaw and Stettler [46] found that markers with distortion and markers without distortion held the same mapping efficiency and that genes may lose the linkage relationship when discarding the distorted markers.

In our integrated map, distorted markers were mainly distributed in LG12. And one or two distorted markers existed in LG4, LG9, LG13 and LG17. These distorted markers did not affect the sequence of marker order and inversely played a good role in linking. Therefore, they were used for map construction.

Compared with co-dominant SSR markers, dominant markers are less capable of covering the map length.[4] Here, we construct a *V. vinifera* genetic map based on SSR markers, and we also added 119 dominant SRAP markers into the *V. vinifera* genome. These SRAP markers, on the basis of SSR markers, not only lengthened the linkage groups, but also increased the marker density of the linkage groups and filled some gaps between SSR markers. We consider that SRAP markers play a role of supplementation in map construction. However, we did not obtain LG11 in the ‘9-22’ map, and LG4, LG8, LG13 were broken into two parts in the integrated map. Further research should focus on new developed EST-SSR and SNP markers from the 12 times grapevine genome sequence (http://www.genoscope.cns.fr/externe/Genome-Browser/Vitis/) and on increasing the number of SRAP markers, so as to improve the quality of the genetic map.

## Conclusions

Here, we created an intraspecific *Vitis vinifera* cross population using ‘87-1’ and ‘9-22’. To construct the parental map and consensus map, which well covered the 19 chromosomes of the *Vitis* genome, 149 F1 individuals were used. Based on SSR markers, comparisons were made between this map and the reference maps. The *V. vinifera* map was complemented by using SRAP markers. This map could be used for QTL mapping, and could also lay the foundation for further studies such as marker-assisted selection.
